# The Effect of TISSEEL^®^ on the Healing Process of Uterine Horn Reanastomosis in an Experimental Animal Model

**DOI:** 10.3390/medicina62020333

**Published:** 2026-02-06

**Authors:** Dimitrios Papageorgiou, Vasilios Pergialiotis, Nikolaos Salakos, Stylianos Kykalos, Kalliroi Goula, Konstantinos Kontzoglou

**Affiliations:** 1 Department of Gynecology, Athens Naval and Veterans Hospital, 11521 Athens, Greece; 2 Laboratory of Experimental Surgery and Surgical Research “N.S. Christeas”, School of Medicine, National and Kapodistrian University of Athens, 11527 Athens, Greece; 3 First Department of Obstetrics and Gynecology, Unit of Gynecologic Oncology, “Alexandra” Hospital, School of Medicine, National and Kapodistrian University of Athens, 11527 Athens, Greece; 4 School of Medicine, National and Kapodistrian University of Athens, 11527 Athens, Greece; 5 Second Department of Propaedeutic Surgery, “Laikon” General Hospital, School of Medicine, National and Kapodistrian University of Athens, 11527 Athens, Greece; 6 Department of Pathology, “Alexandra” Hospital, 11528 Athens, Greece

**Keywords:** fibrin sealant, TISSEEL^®^, tubal reanastomosis, postoperative adhesions, anastomotic healing, neovascularization, collagen deposition, experimental rat model, histologic outcomes, fertility preservation

## Abstract

*Background and Objectives*: Tubal reanastomosis is an alternative option for women seeking fertility after sterilization. Thus, anastomosis healing quality and peri-tubal adhesions play a crucial role. TISSEEL^®^ fibrin sealant may enhance tissue repair and reduce foreign-body reaction. We evaluated the effect of TISSEEL^®^, used alone or with sutures, on anastomotic healing and adhesion formation in a rat uterine horn model. *Materials and Methods*: Thirty female Wistar rats were randomized to Suture, TISSEEL^®^, or Suture + TISSEEL^®^ groups (*n* = 10 each). After bilateral uterine horn transection, reanastomosis was performed with sutures alone, fibrin sealant alone, or combined sutures and sealant. On postoperative day 14, reanastomosis segments were collected for blinded histologic assessment and evaluation of modified Ehrlich–Hunt score parameters (inflammation, fibrosis, neovascularization and collagen production). Intra-abdominal adhesions were also macroscopically assessed. *Results*: Two animals died perioperatively and 56 uterine horns were included in the final analysis (Suture *n* = 18, TISSEEL^®^ *n* = 18, Suture + TISSEEL^®^ *n* = 20). The distribution of inflammation and fibrosis severity grades, as assessed by the modified Ehrlich–Hunt scoring system, did not differ significantly between the study groups (*p* = 0.208 and *p* = 0.652, respectively). In contrast, high-grade neovascularization (grades 3–4) was more common in TISSEEL^®^ groups (77.8% TISSEEL^®^, 80.0% Suture + TISSEEL^®^, 33.3% Suture, *p* = 0.004), while increased collagen deposition was also more common in the TISSEEL^®^ groups (*p* = 0.011), after binary analysis. Severe adhesions were more common in the Suture group (66.7% vs. 11.1% in the TISSEEL^®^ group and 30.0% in the Suture + TISSEEL^®^ group, *p* = 0.037). *Conclusions*: TISSEEL^®^, alone or as an adjunct to sutures, improves neovascularization and collagen production and is associated with milder adhesions without increased inflammation or fibrosis. The use of fibrin sealant TISSEEL^®^ may be a useful tool in tubal reconstructive surgery.

## 1. Introduction

Female sterilization accounts for one-quarter of modern contraceptive use, with 219 million users worldwide. The prevalence of the method is higher in Central and Southern Asia, in Latin America and in the Caribbean, while it remains less common in sub-Saharan Africa and Europe [1,2]. Approximately 18% of women aged 15–49 in the United States undergo female sterilization, a percentage that escalates to about 39% among those aged 40–49, indicating that usage of this method increases with age and parity [3].

Research shows that regret after sterilization occurs regularly and age plays a major role in this phenomenon. Especially, 10% of women mention regret after sterilization. This rate reaches 12.6% among those sterilized between 21 and 30 years old compared to 6.7% among those over 30 years old [4]. The average patient seeking reversal treatment, according to recent series, is a mid-thirties parous woman who has received tubal ligation 4 to 7 years ago, because she wants additional children with her new partner [5,6,7].

Tubal reanastomosis involves microsurgical reconstruction of fallopian tubes that have been previously ligated, in order to restore patency, aiming to achieve natural conception [8]. This procedure allows patients to achieve multiple natural pregnancies while eliminating the need for ovarian stimulation and multiple IVF cycles [6,8,9]. The rate of pregnancy achievement after reanastomosis reaches 60–65% and ectopic pregnancy occurs in 6–7% of cases, making it a suitable treatment for patients who do not require IVF [6].

Fibrin sealants such as TISSEEL^®^ mimic the final stages of blood coagulation through the formation of a temporary fibrin clot, which affects early wound healing responses, including inflammation, neovascularization and fibrosis [10,11]. Research across different tissues shows that fibrin sealants produce opposing effects on tissue repair depending on the particular situation when used in bowel and pelvic microsurgery. The application of commercial fibrin glue to skin regeneration matrices during in vitro experiments resulted in reduced angiogenic and lymphangiogenic gene and protein expression in endothelial and stromal cells [12]. Clinical and experimental studies in visceral surgery demonstrate that fibrin sealant provides excellent sealing capabilities and rapid healing effects. A retrospective cohort study demonstrated that fibrin sealant reduced staple line leaks after sleeve gastrectomy procedures [13]. The use of fibrin sealants has also been used for endoscopic closure of duodenal perforations that occurred during ERCP procedures [14]. The experimental study of TISSEEL^®^ in confined bowel perforation models showed better tissue regeneration with decreased inflammation and fibrosis compared to suture closure [15]. The study used rats to show that fibrin-based fixation of polypropylene mesh produced lower adhesion rates than suture fixation [16]. However, evidence specifically addressing tubal anastomoses is limited and the net impact of fibrin sealants on anastomotic histological healing and peritubal adhesion formation remains unclear.

Fallopian tube reanastomosis using microsurgical techniques remains a crucial fertility preservation procedure which enables women to reverse sterilization and repair tubal damage resulting from iatrogenic or disease–related tubal damage [5,6,7,8,9]. The fallopian tube presents a distinct microenvironment which includes a delicate mucosa with ciliated epithelial cells, a thin muscular layer and a highly vascular serosa that exists in a peritoneal space, where even modest ischemia, edema or fibrous tissue growth can threaten tubal patency [8,16,17]. The combination of microsutures with clips and sealants during surgical tubal repair aims to maximize patency while minimizing tissue injury, ischemia and adhesions’ formation [8,11,16,17].

We therefore conducted an experimental study to evaluate the effect of TISSEEL^®^ on histological healing after tubal anastomosis in Wistar rats, when used either as a suture substitute or as an adjunct to microsuturing. We hypothesized that TISSEEL^®^ would primarily enhance neovascularization and collagen deposition at the anastomotic site and reduce the severity of postoperative adhesions, while not increasing the inflammatory response or fibrosis compared with suturing alone.

## 2. Materials and Methods

### 2.1. Ethical Approval Subsection

The experimental protocol received institutional approval by the Ethics Committee of the Medical School of National and Kapodistrian University of Athens, Greece (Ref. Nο. 620/23 February 2024), after receiving veterinary animal use approval by the Veterinary Service of the Prefecture of Attica, Greece. All surgical procedures and animal care (pre- and postoperative) adhered to European Union Directive 2010/63/EU regarding animal experimentation. The experimental procedures were conducted between March 2025 and April 2025.

### 2.2. Animal Care

Thirty female Wistar rats, procured from the National Centre for Scientific Research “Demokritos” were transferred to the Laboratory of Experimental Surgery “NS Christeas” of the National and Kapodistrian University of Athens, where they were housed in controlled-conditions chambers (humidity 55 ± 5%, temperature 20 ± 1 °C, controlled light levels 12 h day/night cycle) for seven days, before surgical interventions, to acclimate to their new environment. Unlimited access to water and EVIZ 510 food pellets has been provided throughout the duration of the experimental study.

### 2.3. Study Design, Randomization, Blinding and Sample Size

Animals were allocated to the three treatment groups (1:1:1, 10 rats per group) by simple random allocation. An investigator not involved in the surgical procedures prepared sequentially numbered, sealed envelopes containing the group assignment, shuffled them before the start of the experiment and the envelopes were opened immediately before surgery. Thus, allocation was concealed until assignment. Because the interventions were readily apparent, the operating surgeon was not blinded. However, histopathological assessment was performed blinded to group allocation.

Sample size was determined a priori using the resource equation method as described by Charan and Kantharia [18]. According to this, our study required at least seven animals for each group. We enrolled ten animals per group to handle expected losses during surgery and tissue preparation, which would maintain the predetermined analysis threshold, an important consideration for interpreting non-significant findings.

### 2.4. Fibrin Sealant

The fibrin sealant TISSEEL^®^ (Baxter Healthcare Corporation, Deerfield, IL, USA) is a biocompatible product that combines two sterile, deep-frozen coagulation solutions: thrombin and sealer protein. The preloaded syringe contains two separate chambers for each solution. The thrombin solution contains thrombin and calcium chloride from pooled human plasma, whereas the sealer protein solution contains synthetic aprotinin, factor XIII and fibrinogen [12]. TISSEEL^®^ mimics the final stages of blood coagulation and forms a stable thrombus when applied. Thrombin transforms fibrinogen into fibrin monomers, which then assemble into a fibrin thrombus with coagulation factor XIII, while aprotinin stabilizes the structure to prevent degradation. The thrombin and sealer protein solutions need to be heated at 37 °C and mixed in a single syringe before use [19,20].

### 2.5. Experimental Protocol and Surgical Interventions

Anesthesia was achieved via xylazine hydrochloride (5 mg/kg) and ketamine (60 mg/kg) intraperitoneal administration. Xylazine is a clonidine analog and α-2 adrenergic receptor agonist. Ketamine is used to induce and maintain anesthesia by causing sedation and analgesia [15]. After proper anesthesia, antisepsis and drape of the anterior abdominal wall were performed with povidone iodine solution, alcohol and sterile drapes. A vertical midline incision of about 4 cm followed, starting right below the xiphoid process and ending about 1 cm above the external genitalia. Upon accessing the peritoneal cavity, the uterine horns were identified bilaterally.

**Figure 1:**
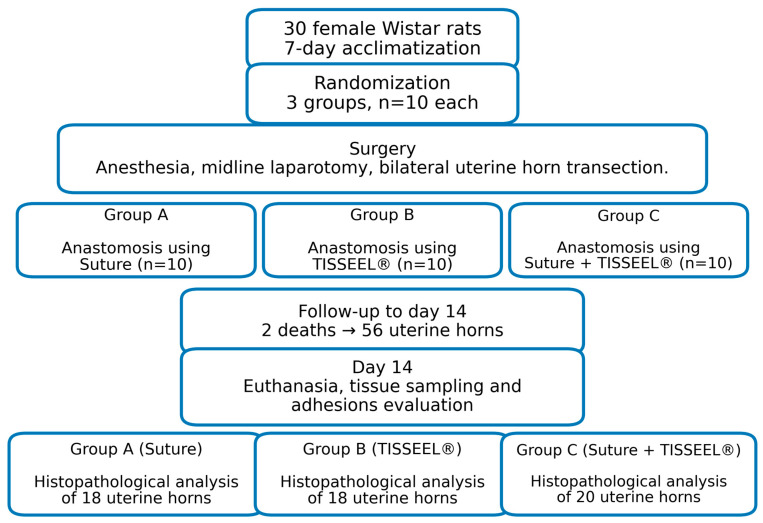
Flow diagram of animal randomization and experimental design.

The rats were randomized into three different treatment groups of ten (Figure 1). A segment about 0.5 cm long was removed from the distal end of each horn, followed by an end-to-end anastomosis. The uterine horns in Group Suture were anastomosed bilaterally with a 4-0 synthetic, monofilament, absorbable surgical suture made of polyglyconate. In Group TISSEEL^®^, bilateral anastomosis was performed by topical application of about 0.4 mL of TISSEEL^®^. In Group Suture + TISSEEL^®^, the anastomosis was done, bilaterally, using a combination of 4-0 synthetic, monofilament, absorbable surgical suture made of polyglyconate and TISSEEL^®^. In order to facilitate smooth re-approximation, when TISSEEL^®^ was employed, the anastomosis was performed by approaching the ends of the uterine horn over a silicone catheter inserted through a small incision into the proximal segment of the horn. TISSEEL^®^ was applied to the serosal surface to prevent intraluminal entrance and blockage. The catheter stayed in place for at least two minutes. Abdomen and skin closure were performed with 2-0 multifilament 910-polyglactin absorbable Novosyn continuous suture. Subcutaneous tramadol administration, at a dose of 0.01 mg/kg, was used for analgesia during the postoperative period.

The animals were euthanized on the 14th day post-op. After euthanasia, an excision of the anastomotic site together with almost 1 cm proximal and distal segments of the uterine horns was performed. Tissue samples were then sent for histopathological evaluation. During relaparotomy, two researchers, blinded to group allocation, macroscopically assessed intra-abdominal adhesions. The severity and area of adhesions were evaluated with a widely used scoring system (Table 1) that rates adhesions from none (degree 0) to thick adhesions with planar attachment that cover 75–100% of the initial injured area (degree 4) [21].

### 2.6. Histopathology Evaluation

The removed specimens were then flushed with saline solution to remove blood from the surface and were placed in formalin. The specimens underwent standard processing before being embedded in paraffin and sectioned longitudinally to display the anastomotic line and lumen in the same plane.

Hematoxylin—eosin stain was used to study inflammatory infiltrate, fibroblast proliferation and angiogenic capillaries and collagen deposition. The modified, widely used Ehrlich–Hunt quantitative scoring system was used to assess histologic healing. Inflammation, fibrosis, neovascularization and collagen production have been assessed using the aforementioned scale as follows: 0: no evidence of the parameter, 1: occasional evidence of the parameter, 2: light scattering or low density, 3: abundant evidence but separated, and 4: confluent cells or fibers, indicating high density [12,22].

Pathology evaluation was blinded. Slides were coded by an investigator and the pathologist was uninformed of the group assignment.

### 2.7. Statistical Analysis

SPSS version 25.0 for Windows (SPSS Inc., Chicago, IL, USA) was used to do all analyses. Intention-to-treat analysis was conducted. Given the ordinal nature of the histological scores and the non-normal distribution of the data, categorical and ordinal variables were summarized using absolute and relative frequencies (numbers and percentages). Continuous animal characteristics were reported as median (range) values. Comparisons between groups for ordinal histological parameters (inflammation, fibrosis, neovascularization, and collagen production scores) were performed using the chi-square test. When appropriate, histological scores were dichotomized into low (grades 1–2) and increased (grades 3–4) categories and between-group differences were assessed using chi-square analysis. Comparisons of continuous variables between more than two groups were performed using the Kruskal–Wallis test. For adhesion severity, the primary analysis used the full five-grade macroscopic classification (0–4). In addition, a prespecified sensitivity analysis collapsed the five grades into three clinically meaningful categories (no adhesions, thin/mild adhesions, thick/severe adhesions) to mitigate sparse categories and facilitate interpretation. Analogous binary collapsing (grades 1–2 vs. 3–4) was applied to selected histologic parameters as a secondary analysis. All tests were two-sided, and *p* < 0.05 was considered statistically significant. Secondary sensitivity analyses were interpreted cautiously and considered supportive.

## 3. Results

### 3.1. Animal Characteristics

Our research included a total of 30 female Wistar rats distributed into three experimental groups (Suture *n* = 20, TISSEEL^®^ *n* = 20, Suture + TISSEEL^®^ *n* = 20). One animal of the Group Suture died perioperatively and one animal of Group TISSEEL^®^ died during the first day post-op. Therefore, 56 uterine horn specimens were included in the final histological analysis (Suture *n* = 18, TISSEEL^®^ *n* = 18, Suture + TISSEEL^®^ *n* = 20).

The median age (16 weeks, range 15–17 weeks) did not differ among groups (Kruskal–Wallis, *p* = 0.593). The groups showed a statistically significant difference in their weight according to the Kruskal–Wallis test (*p* < 0.001). The Suture group had the highest median weight at 281 g (range 271–297 g), followed by the TISSEEL^®^ group at 271 g (range 248–285 g). The Suture + TISSEEL^®^ group had the lowest median weight at 257 g (range 233–284 g). Although this difference was found, there was no indication that weight affected the histological parameters, as no relevant correlations were observed in the individual analyses.

### 3.2. Histological Analysis

#### 3.2.1. Inflammation

Inflammation was assessed on the Ehrlich–Hunt five-point scale (grades 0–4). In the Suture group, grades 1, 2, 3 and 4 were recorded in 3 (16.7%), 6 (33.3%), 7 (38.9%) and 2 (11.1%) specimens, respectively. In the TISSEEL^®^ group, the corresponding frequencies were 6 (33.3%), 6 (33.3%), 4 (22.2%) and 2 (11.1%), while in the Suture + TISSEEL^®^ group, grades 2 were recorded in 10 (50%), 3 in 8 (40%) and 4 in 2 (10%) samples, with no sample showing grade 0 or 1.

No statistically significant differences were observed in the distribution of inflammation severity grades between the three experimental groups (Pearson χ^2^ = 8.554, df = 6, *p* = 0.208). Despite the absence of statistical significance, it was observed that the Suture group exhibited the highest rate of moderate to severe inflammation (grades 3–4) (50%), while the TISSEEL^®^ group exhibited the mildest inflammatory reaction, with 67% of samples in grades 1–2, suggesting that the use of TISSEEL^®^ does not enhance inflammation and may contribute to a milder tissue reaction (Table 2 and Table 3) (Supplementary File S1).

#### 3.2.2. Fibrosis

No statistically significant differences were observed in the distribution of fibrosis severity grades between the three experimental groups (Pearson χ^2^ = 1.125, df = 2, *p* = 0.652). In the Suture group, grade 1 was recorded in 10 (55.6%) specimens and grade 2 in 8 (44.4%), while in the TISSEEL^®^ group, the percentages were the same (10 and 8, respectively). The Suture + TISSEEL^®^ group showed 14 (70%) grade 1 and 6 (30%) grade 2. Higher fibrosis severity grades (grades 3–4) were not observed in any group, indicating that neither suture nor TISSEEL^®^ nor their combination induces a strong fibrotic reaction (Table 2 and Table 3) (Supplementary File S1).

#### 3.2.3. Neovascularization

**Figure 2:**
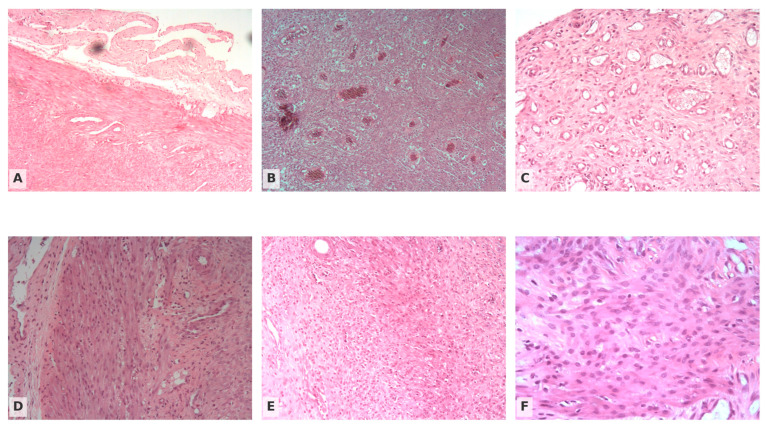
Histologic appearance of uterine horn anastomoses on postoperative day 14 (hematoxylin—eosin stain). (**A**) Suture group, 10× magnification, showing a relatively homogeneous fibrous band at the anastomotic site with loosely organized collagen and a few small vessels. (**B**) TISSEEL group, 20× magnification, showing densely cellular fibrous tissue with more compact collagen bundles and numerous small vascular lumina, indicating increased collagen deposition and neovascularization. (**C**) Suture + TISSEEL group, 10× magnification, showing moderately dense collagenous tissue with several small vessels, representing an intermediate pattern between the Suture and TISSEEL groups. (**D**) Suture group, 20× magnification, showing fibroblast-rich granulation tissue with loosely arranged collagen fibers and scarce vessels. (**E**) TISSEEL group, 20× magnification, showing plump fibroblasts and endothelial cells within collagenizing granulation tissue rich in newly formed capillaries. (**F**) Suture + TISSEEL group, 20× magnification, showing fibroblast-rich granulation tissue with moderately organized collagen fibers and scattered small vessels, again intermediate between the Suture and TISSEEL groups.

Neoangiogenesis altered between groups, with significant statistical differentiation (Pearson χ^2^ = 24.028, df = 6, *p* < 0.001). In the Suture group, grades 1 to 4 were recorded in 4 (22.2%), 8 (44.4%), 2 (11.1%) and 4 (22.2%) uterine horns, respectively, with a total of 66.6% belonging to low angiogenic activity (grades 1–2). In the TISSEEL^®^ group, the picture was reversed: only 2 (11.1%) samples were grade 1 and 2 (11.1%) were grade 2, while 8 (44.4%) and 6 (33.3%) were grade 3 and 4, respectively, with 77.7% of the samples showing high vascularization (grades 3–4) (Figure 2). The Suture + TISSEEL^®^ group showed the most pronounced neovascularization profile of all: 4 (20%) grade 1 samples, no grade 2 samples, 2 (10%) grade 3 samples and an impressive 14 (70%) grade 4 reactions (Table 2) (Supplementary File S1).

Binary analysis (low angiogenesis 1–2 vs. high angiogenesis 3–4) confirmed this significant difference (Pearson χ^2^ = 11.089, df = 2, *p* = 0.004) reaction (Table 3) (Supplementary File S1).

#### 3.2.4. Collagen Production

Regarding collagen production, the Suture group contained 6 (33.3%) samples rated as 1 and 12 (66.7%) samples rated as 2 but no samples received a grade 3 or higher rating. The Suture + TISSEEL^®^ group contained 6 (30%) samples rated as 1 and 14 (70%) samples rated as 2. The TISSEEL^®^ group displayed 4 (22.2%) grade 1, 10 (55.6%) grade 2 and 4 (22.2%) grade 3 ratings. The multilevel statistical analysis showed minimal variations between groups and a difference that approached but did not reach statistical significance (Pearson χ^2^ = 9.186, df = 4, *p* = 0.055). When results were dichotomized into low collagen production (grades 1–2) vs. high collagen production (grades 3–4), binary analysis showed a statistically significant difference (Pearson χ^2^ = 9.094, df = 2, *p* = 0.011), with the TISSEEL^®^ group showing the highest proportion of increased collagen deposition reaction (Table 2 and Table 3 and Figure 2) (Supplementary File S1).

### 3.3. Adhesion Formation

Statistical analysis of the full classification (five-grade scale) showed no significant difference (Pearson χ^2^ = 11.148, df = 8, *p* = 0.193). Specifically, the Suture group showed 3 (33.3%) thin adhesions and 6 (66.7%) thick adhesions. The TISSEEL^®^ group showed 2 (22.2%) no adhesions, 6 (66.7%) thin and only 1 (11.1%) thick adhesive band. The Suture + TISSEEL^®^ group showed 7 (70%) thin and 3 (30%) thick adhesions.

However, the prespecified sensitivity analysis, in which adhesions were classified in three clinically meaningful categories: no adhesions (grade 0), thin/mild adhesions (grades 1–2) and thick/severe adhesions (grades 3–4), revealed a marginally statistically significant difference between the groups (Pearson χ^2^ = 9.578, df = 4, exact *p* = 0.037), allowing for a clearer comparative assessment (Table 4) (Supplementary File S1).

In the Suture group, the highest proportion of severe adhesions was observed, as 6 of the 9 samples (66.7%) showed thick adhesions, while only 3 (33.3%) showed thin ones. In contrast, the TISSEEL^®^ group showed the lowest frequency of severe adhesions, with only 1 of the 9 samples (11.1%) showing a thick adhesive band. The remaining 8 samples (88.9%) showed either thin or no adhesions. The Suture + TISSEEL^®^ group presented an intermediate profile, with 3 of 10 samples (30%) showing thick adhesions and the remaining 7 samples (70%) thin (Table 4) (Supplementary File S1).

## 4. Discussion

### 4.1. Principal Findings

In this rat experimental study, the use of suture, TISSEEL^®^ fibrin sealant and their combination was associated with distinct healing patterns. No statistically significant differences in inflammatory response and fibrosis were observed between the three groups. In contrast, neovascularization was significantly more intense in the suture + TISSEEL^®^ group. Collagen production showed a marginally significant difference in the ordinal score analysis and reached statistical significance when the data were dichotomized, with the TISSEEL^®^ group showing the highest frequency of increased collagen deposition. Regarding postoperative adhesions, the primary five-grade analysis did not demonstrate a significant difference between groups. However, binary analysis suggested a shift toward less severe adhesions, with the suture group showing the highest proportion of thick adhesions and the TISSEEL^®^ group the most favorable distribution (more absent or thin adhesions).

Overall, these findings suggest that adding TISSEEL^®^, either alone or as an adjunct to microsuturing, may qualitatively influence early anastomotic healing toward a profile characterized by enhanced angiogenesis and collagen formation, without a concomitant increase in fibrosis or marked inflammation. Improved microvascularization at the anastomosis site is critical for maintaining the viability of the epithelial and muscular layer, parameters that are directly linked to ovum fertilization and embryo transport. Thus, this profile may lead to maintaining tubal elasticity and patency and reduce the risk of postoperative adhesions, factors critical for future fertility and reproductive function and the risk of ectopic pregnancy [6,23].

### 4.2. Comparison to Existing Literature

#### 4.2.1. Inflammation

The absence of statistically significant differences in inflammation between groups, with the suture group showing slightly more moderate-to-severe inflammation (grades 3–4 in 50% of the tubes), but without a statistically important difference (*p* = 0.208), suggests that the use of TISSEEL^®^ does not trigger an excessive acute or chronic inflammatory response compared with mechanical suture trauma. This is consistent with the general experience with the use of fibrin glue as a biological sealant, where good biocompatibility and the absence of significant additional inflammatory reaction have been documented in various surgical fields [12,15,20]. Comparable findings have also been reported in a controlled rat peritoneal model, where a fibrin glue barrier did not significantly affect the histological inflammatory reaction or peritoneal healing compared with controls [24]. The mild and relatively uniform inflammatory response observed in all groups can be considered a normal consequence of surgical intervention and not an undesirable effect of the respective material. This is particularly important because the use of biological or synthetic materials in pelvic surgery is often accompanied by concern for increased local inflammation, which may trigger adhesion formation. The literature confirms that the type, intensity and duration of the inflammatory response are decisive factors in the conversion of normal healing to pathological symphysiogenesis [25,26,27].

#### 4.2.2. Fibrosis

Fibrosis did not differ significantly between the three experimental groups, with most specimens exhibiting low fibrosis grades (grade 1 in 55–70% of tubes in each group). This is particularly interesting because increased fibrosis could theoretically be the price of more intense healing, especially when biological sealants are used. However, available experimental data suggest that fibrin sealants can be histologically neutral in serosal environments. In a rabbit intraperitoneal model, fibrin sealant fixation did not exert a significant histologic effect, including fibrosis, compared with alternative fixation approaches [28].

These findings are also consistent with prior experimental work. When used as a bioabsorbable closure material, fibrin glue has been associated with a balanced healing profile characterized by adequate reconstruction without excessive scarring of the tubal wall and improved tissue organization [20,29,30,31]. TISSEEL^®^ has been reported to promote tissue healing in nerve, bone and peritoneal tissue models by preventing hypertrophic fibrosis and producing less dense fibrotic tissue than nonabsorbable scaffolds [31]. Ιt has been found that fibrin glue can, depending on the dosage and model, reduce connective tissue deposition and the severity of adhesions, possibly acting as a temporary biological barrier [16,26,32].

From a reproductive perspective, avoiding excessive fibrosis and inflammation when using TISSEEL^®^, either alone or in combination with suturing, is particularly important. Both processes have been associated with luminal obstruction and dense adhesion formation, which can contribute to infertility and ectopic pregnancy complications [20,29].

#### 4.2.3. Collagen Production

Experimental wound-healing models indicate that tissues exposed to fibrin sealants may produce more collagen, as sealants function not only as hemostatic agents but also promote tissue regeneration [12,20,33]. Research studies on fibrin-based scaffolds have shown that these materials stimulate fibroblasts to produce type I and III collagen while supporting fast extracellular matrix organization and maturation and reducing the possibility of abnormal scarring [33,34,35,36].

On the other hand, other studies have reported reduced collagen deposition when specific fibrin sealant protocols have been used. Especially, Eby et al. studied the impact of fibrin sealant on guinea pig skin healing, revealing that fibrin-treated wounds exhibited a decrease in both inflammation and collagen deposition, alongside achieving complete reepithelialization. Their data indicate that, in this specific skin model, fibrin sealant inhibits collagen deposition and scarring [37].

In our study, the TISSEEL^®^ group showed increased collagen deposition without an accompanying increase in fibrosis development or thick adhesion formation. This leaves open the possibility that enhanced collagen production reflects more qualitative wall remodeling, which results in stronger anastomoses and better-organized extracellular matrix structure, instead of leading to pathological scarring. The variability across experimental models and protocols suggests that outcomes after fibrin sealant application depend on multiple factors, including sealant type, dose, follow-up duration and target tissue.

#### 4.2.4. Neovascularization

In our study, neovascularization was the most clearly differentiated parameter among the three experimental groups. The Suture + TISSEEL^®^ group demonstrated the most pronounced vascular response, with grade 4 neovascularization in 70% of the specimens (14/20), while in the Suture group, two-thirds of the samples (12/18, 66.7%) were limited to low grades. The TISSEEL^®^ group showed an intermediate but clearly increased neovascularization profile.

Our results align with existing research on fibrin products in experimental models and trials. Reviews of fibrous substrates in wound healing highlight that the fibrin clot and its derivatives act as a three-dimensional biosubstrate for endothelial cells and fibroblasts, facilitating the formation of new capillaries and the organization of granulation tissue, with a direct consequence of enhancing neovascularization in various skin and soft tissue models [20,33]. In line with the above, fibrin glue application to the laser-resurfaced skin model resulted in better vascularization, but it caused a slight delay in reepithelialization, suggesting that new that the fibrin matrix favors the early vascular phase of healing [37]. Recent research on the effect of platelet-rich fibrin and composite fibrin matrices on wound healing confirms that the fibrin matrix can improve angiogenesis through local release of growth factors, creating an environment of high vascular activity that favors tissue regeneration [38,39]. The study by Gerogiannis et al. showed that rat ileoileal anastomoses treated with fibrin glue developed enhanced microvascularity compared to sutured anastomoses, but this neovascularization did not improve reanastomosis mechanical strength [40]. Similarly, Stergios et al. reported that TISSEEL^®^ application during colorectal anastomosis in rats under hyperglycemic stress conditions resulted in improved neovascularization [12].

Nevertheless, the effects of fibrin sealants on vascularization are not uniform and may depend on the specific product, dose, layer thickness, and target tissue. In contrast to the studies noted above, experimental work in free-skin flaps suggests that TISSEEL^®^ does not significantly influence short-term revascularization or flap survival, implying a neutral effect in that context [41].

The enhanced neovascularization in the Suture + TISSEEL^®^ group, compared to both suture and TISSEEL^®^ groups, suggests that the mechanical stabilization of the tissue-target in combination with the fibrous substrate creates a microenvironment where angiogenesis is favorable. A more stable approximation of the anastomosed surfaces may allow the fibrin material to function as an organized matrix to which endothelial cells can adhere, thereby accelerating neovascularization, as has been described for other fibrous scaffolds [33,42].

#### 4.2.5. Adhesions Formation

Our study shows that TISSEEL^®^, whether used alone or in conjunction with sutures, was associated with reduced adhesion severity compared with suturing alone. In binary analysis, the Suture group exhibited the highest proportion of thick adhesions (67%). In contrast, the TISSEEL^®^ group demonstrated the lowest incidence of severe adhesions (11%), with the Suture + TISSEEL^®^ group showing an intermediate pattern.

Peritoneal adhesions are formed when opposing, injured serous surfaces remain in prolonged contact within a fibrin-rich exudate, combined with inadequate local fibrinolysis and excessive inflammatory and fibroblastic responses [26,27,32]. Within this context, the exogenous fibrin clot created by TISSEEL^®^ could theoretically raise concerns about increased adhesion production. However, our results, in conjunction with previous studies [16,21,26,32], support the hypothesis that fibrin glue functions as a biological barrier between tissue surfaces in the initial phases of healing, perhaps diminishing the contact between wounded surfaces, a critical process in adhesion formation. The absence of increased fibrosis and the lack of excessive inflammation in the TISSEEL^®^ groups in our study further support this interpretation.

These observations are consistent with the available data on adhesion prevention strategies in abdominal and pelvic surgery. Recent reviews suggest that careful surgical management (atraumatic technique, careful hemostasis, minimizing foreign material) combined with the use of bioabsorbable barriers can reduce both the incidence and severity of adhesions, although no measure completely eliminates the problem [16,25,27,32]. Notably, the experimental literature on fibrin sealants in gynecological models is heterogeneous. While some studies report reduced adhesions, others report no measurable benefit, highlighting the influence of injury severity, surgical technique and timing of assessment [43,44].

Experimental anti-adhesion products in rat models, including bioabsorbable membranes, gels and fibrin-based materials, have shown a reduction in adhesion scores [16,21]. Our data suggest that TISSEEL^®^ may exhibit similar “barrier behavior” at the site of anastomosis, while simultaneously supporting a more organized healing pattern.

Clinically, this adhesion pattern is of particular relevance to tubal surgery. Dense peri-fallopian adhesions and large-area serous adhesions are closely associated with impaired tubal motility, tubo-ovarian anatomy distortion, infertility and an increased risk of ectopic pregnancy [23,29,32]. The prevalence of thick adhesions in the Suture group versus the prevalence of thin or absent adhesions in the TISSEEL^®^ group suggests that suturing alone may predispose to less favorable conditions compared with fibrin-assisted repair in this model.

### 4.3. Clinical Significance of the Study

The present experimental model provides pathophysiological data that are directly relevant to tubal reconstructive surgery. Tubal recanalization is an important fertility-preservation option for women who regret sterilization. Reported pregnancy rates after tubal recanalization are approximately 60–65%, resulting in spontaneous pregnancies and reducing the need for IVF treatments [5,6,7,8,9,23]. In this context, any technique or adjunct that supports orderly healing of the tubal wall while minimizing luminal compromise and peritubal adhesions may have clinical value.

Our findings suggest that TISSEEL^®^ may improve anastomotic healing when used alone or with sutures by promoting neovascularization and collagen deposition without increasing inflammation or fibrosis. This pattern is particularly desirable for the fallopian tube, where epithelial viability, preservation of the muscularis, and a compliant non-fibrotic wall are critical for ovum transport and early embryo transit [6,8,9]. The lower frequency of thick adhesions in the TISSEEL^®^ group supports the hypothesis that fibrin sealants can function as short-term biological barriers that facilitate tissue recovery with minimal postoperative adhesion formation [16,20,26,32].

From a practical surgical standpoint, these data reinforce the rationale for exploring fibrin sealant as an adjunct in tubal reanastomosis, particularly in minimally invasive approaches where extensive microsuturing is technically demanding. The application of TISSEEL^®^ in gynecological and visceral surgery has demonstrated better hemostasis, without a consistent signal for increased adhesion formation [12,13,15,20,45]. If the histological advantages observed in this rat model translate into improved tubal patency, lower ectopic pregnancy rates and reduced peri-tubal adhesions, fibrin sealant–assisted reanastomosis could become a valuable alternative for selected patients who are suitable candidates for tubal surgery rather than primary IVF [6,8,9,17,23,45].

### 4.4. Study Strengths and Limitations

This study has several methodological strengths. First, it examines TISSEEL^®^ effects on tubal reanastomosis healing and adhesion formation using a well-established Wistar rat uterine horn model that is widely used for anastomosis and anti-adhesion research. Second, the study design was balanced, with three experimental groups and random allocation. All procedures were performed using standardized surgical and anesthesia protocols in homogeneous animal cohorts housed under controlled conditions. The evaluation of healing was carried out through two methods, which consisted of blinded macroscopic assessment of adhesion severity and blinded histological examination using the modified Ehrlich–Hunt system. Finally, statistical processing combined ordinal analysis with binary tests to detect fine differences between healing patterns and adhesion severity.

Several limitations should be considered when evaluating our research results. First, our study uses the uterine horn of healthy Wistar rats as a reanastomosis model to investigate mechanisms related to the human fallopian tubes. Although rat models have been widely used to investigate the healing of anastomoses and anti-adhesion strategies, there are major anatomical, physiological and hormonal differences between the uterine horns of rats and human fallopian tubes, which may influence the healing response and adhesion formation [20,29,30,31]. In addition, the animals did not reproduce common clinical contexts such as prior pelvic inflammation, endometriosis and scarring, which limits direct extrapolation to clinical microsurgical tubal reanastomosis. Second, the follow-up period was limited to 14 days. This time frame reflects early and intermediate phases of healing but does not capture long-term results regarding healing process quality. Previous research in bowel and soft tissue models shows that the initial histological outcome is not always associated with long-term clinical benefit [20,37,40]. Finally, in our experimental model, we used only a single fibrin sealant (TISSEEL^®^) in a fixed dosage and followed a specific technique. Different concentrations of thrombin, layer thicknesses, application methods or alternative fibrin-based products might produce distinct inflammatory, angiogenic and fibrotic responses [10,11,12,33,34,35,36]. Therefore, the generalizability of our findings to other products and protocols remains limited.

## 5. Conclusions

In this experimental model, the effect of TISSEEL^®^ fibrin sealant, alone or combined with sutures, has been assessed and compared to suturing alone, in order to evaluate the healing of uterine horns and adhesion formation in the Wistar rat model.

Our results indicate that TISSEEL^®^ was associated with enhanced neovascularization and increased collagen deposition, without an accompanying increase in inflammation or fibrosis, and with a more favorable adhesion profile compared with suturing alone. Overall, the findings suggest that TISSEEL^®^ may promote a more organized healing of the uterine horn wall, which is theoretically important for maintaining normal function. However, this is an animal study with limited follow-up and no direct assessment of tubal patency or fertility. Therefore, the results should be interpreted with caution and confirmed in larger, longer-term experimental studies and clinical trials before definitive conclusions can be drawn for routine surgical practice.

## Figures and Tables

**Table 1:** Adhesion severity scoring system used for postoperative macroscopic evaluation.

Degree	Description	
	Adhesion Severity	Adhesion Area
**0**	No adhesions	No adhesions
**1**	Thin filmy adhesion	25% of initial injured area
**2**	More than one thin adhesion	25–50% of initial injured area
**3**	Thick adhesion with focal point	50–75% of initial injured area
**4**	Thick adhesion with planar attachment	75–100% of initial injured area

**Table 2:** Distribution of postoperative day 14 histological grades by treatment group using the modified Ehrlich–Hunt scoring system.

Inflammation Score	Group Suture (*n* = 18) *	Group TISSEEL^®^ (*n* = 18) *	Group Suture + TISSEEL^®^ (*n* = 20) *	*p*-Value *
**0**	0	0	0	0.208
**1**	3	6	0	
**2**	6	6	10	
**3**	7	4	8	
**4**	2	2	2	
**Fibrosis Score**				
**0**	0	0	0	0.652
**1**	10	10	14	
**2**	8	8	6	
**3**	0	0	0	
**4**	0	0	0	
**Neovascularization Score**				
**0**	0	0	0	<0.001
**1**	4	2	4	
**2**	8	2	0	
**3**	2	8	2	
**4**	4	6	14	
**Collagen Production Score**				
**0**	0	0	0	0.055
**1**	6	4	6	
**2**	12	10	14	
**3**	0	4	0	
**4**	0	0	0	

* *n* denotes the number of uterine horn specimens available for histological analysis in each group. Between-group comparisons were performed using Pearson’s chi-square test.

**Table 3:** Binary analysis of Ehrlich–Hunt histologic parameters (low vs. increased) at postoperative day 14.

Parameter	Binary Category ^1^	Group Suture (*n* = 18)	Group TISSEEL^®^ (*n* = 18)	Group Suture + TISSEEL^®^ (*n* = 20)	*p*-Value (Pearson χ^2^)
**Inflammation**	Low	9 (50%)	12 (66.7%)	10 (50%)	0.503
	Increased	9 (50%)	6 (33.3%)	10 (50%)	
**Fibrosis**	Low	18 (100%)	18 (100%)	20 (100%)	NA ^2^
	Increased	0 (0%)	0 (0%)	0 (0%)	
**Neovascularization**	Low	12 (66.7%)	4 (22.2%)	4 (20%)	0.004
	Increased	6 (33.3%)	14 (77.8%)	16 (80%)	
**Collagen Production**	Low	18 (100%)	14 (77.8%)	20 (100%)	0.011
	Increased	0 (0%)	4 (22.2%)	0 (0%)	

Abbreviations: NA: Not available. ^1^ Low = Ehrlich–Hunt grades 1–2, Increased = grades 3–4 for each parameter. ^2^ No χ^2^ test computed for fibrosis because the binary variable was constant (all cases with low fibrosis).

**Table 4:** Postoperative day 14 adhesion severity by group (binary analysis) *.

Adhesion Pattern	Group Suture (*n* = 9) ^1^	Group TISSEEL^®^ (*n* = 9) ^1^	Group Suture + TISSEEL^®^ (*n* = 10) ^1^	*p*-Value
**No adhesions**	0 (0%)	2 (22.2%)	0 (0%)	0.037
**Thin/mild adhesions**	3 (33.3%)	6 (66.7%)	7 (70%)	
**Thick/severe adhesions**	6 (66.7%)	1 (11.1%)	3 (30%)	

* Macroscopic adhesion grades (0–4) were collapsed into three categories: no adhesions (grade 0), thin/mild adhesions (grades 1–2), and thick/severe adhesions (grades 3–4). ^1^ Group sizes (*n*) in this table refer to the number of surviving animals available for macroscopic adhesion assessment on postoperative day 14.

## Data Availability

All data are available upon reasonable request.

## Supplementary Materials

The following supporting information can be downloaded at: https://www.mdpi.com/article/10.3390/medicina62020333/s1, File S1: Statistical Analysis.

## Abbreviations

The following abbreviations are used in this manuscript:

IVF	In Vitro Fertilization
ERCP	Endoscopic Retrograde CholangioPancreatography
