# Supplementary material for: The Effect of TISSEEL® on the Healing Process of Uterine Horn Reanastomosis in an Experimental Animal Model
Source: Medicina (Kaunas). 2026 Feb 6;62(2):333. doi: 10.3390/medicina62020333 (PMC12942363; doi:10.3390/medicina62020333)
Supplement: Supplementary file 1 [file medicina-62-00333-s001.zip › medicina-4024494-File S1.pdf]

Group * Inflammation

Crosstab

Count

		Inflammation				
		1	2	3	4	Total
Group	SUTURE	3	6	7	2	18
	SUTURE+TISSEEL	0	10	8	2	20
	TISSEEL	6	6	4	2	18
Total		9	22	19	6	56

Chi-Square Tests

	Value	df	Asymptotic Significance (2-sided)	Exact Sig. (2-sided)
Pearson Chi-Square	8.554[a]	6	.200	**.208**
Likelihood Ratio	11.026	6	.088	.138
Fisher-Freeman-Halton Exact Test	8.984			.158
N of Valid Cases	56			

a. 6 cells (50.0%) have expected count less than 5. The minimum expected count is 1.93.

Group * Fibrosis

Crosstab

Count

		Fibrosis		
		1	2	Total
Group	SUTURE	10	8	18
	SUTURE+TISSEEL	14	6	20
	TISSEEL	10	8	18
Total		34	22	56

Chi-Square Tests

	Value	df	Asymptotic Significance (2-sided)	Exact Sig. (2-sided)
Pearson Chi-Square	1.125[a]	2	**.570**	.652
Likelihood Ratio	1.145	2	.564	.652
Fisher-Freeman-Halton Exact Test	1.165			.652
N of Valid Cases	56			

a. 0 cells (0.0%) have expected count less than 5. The minimum expected count is 7.07.

Group * Neovascularization

Crosstab

Count

		Neovascularization				
		1	2	3	4	Total
Group	SUTURE	4	8	2	4	18
	SUTURE+TISSEEL	4	0	2	14	20
	TISSEEL	2	2	8	6	18
Total		10	10	12	24	56

Chi-Square Tests

	Value	df	Asymptotic Significance (2-sided)	Exact Sig. (2-sided)
Pearson Chi-Square	24.028[a]	6	<.001	**<.001**
Likelihood Ratio	24.914	6	<.001	<.001
Fisher-Freeman-Halton Exact Test	21.450			<.001
N of Valid Cases	56			

a. 9 cells (75.0%) have expected count less than 5. The minimum expected count is 3.21.

Group * Collagen

Crosstab

Count

		Collagen			
		1	2	3	Total
Group	SUTURE	6	12	0	18
	SUTURE+TISSEEL	6	14	0	20
	TISSEEL	4	10	4	18
Total		16	36	4	56

Chi-Square Tests

	Value	df	Asymptotic Significance (2-sided)	Exact Sig. (2-sided)
Pearson Chi-Square	9.186[a]	4	.057	**.055**
Likelihood Ratio	9.843	4	.043	.065
Fisher-Freeman-Halton Exact Test	6.722			.111
N of Valid Cases	56			

a. 3 cells (33.3%) have expected count less than 5. The minimum expected count is 1.29.

Group * Inflammation_binary

Crosstab

Count

		Inflammation_binary		
		Low production	Increased production	Total
Group	SUTURE	9	9	18
	SUTURE+TISSEEL	10	10	20
	TISSEEL	12	6	18
Total		31	25	56

Chi-Square Tests

	Value	df	Asymptotic Significance (2-sided)	Exact Sig. (2-sided)
Pearson Chi-Square	1.373[a]	2	**.503**	.534
Likelihood Ratio	1.395	2	**.498**	.534
Fisher-Freeman-Halton Exact Test	1.388			.534
N of Valid Cases	56			

a. 0 cells (0.0%) have expected count less than 5. The minimum expected count is 8.04.

Group * Fibrosis_binary

Crosstab

Count

		Fibrosis_binary	
		1.00	Total
Group	SUTURE	18	18
	SUTURE+TISSEEL	20	20
	TISSEEL	18	18
Total		56	56

Chi-Square Tests

	Value
Pearson Chi-Square	.[a]
N of Valid Cases	56

a. No statistics are computed because Fibrosis_binary is a constant.

Group * Neovascularizatio_binary

Crosstab

Count

		Neovascularizatio_binary		
		1.00	2.00	Total
Group	SUTURE	12	6	18
	SUTURE+TISSEEL	4	16	20
	TISSEEL	4	14	18
Total		20	36	56

Chi-Square Tests

	Value	df	Asymptotic Significance (2-sided)	Exact Sig. (2-sided)
Pearson Chi-Square	11.089[a]	2	**.004**	.004
Likelihood Ratio	10.997	2	.004	.006
Fisher-Freeman-Halton Exact Test	10.429			.006
N of Valid Cases	56			

a. 0 cells (0.0%) have expected count less than 5. The minimum expected count is 6.43.

Group * Collagen_binary

Crosstab

Count

		Collagen_binary		
		1.00	2.00	Total
Group	SUTURE	18	0	18
	SUTURE+TISSEEL	20	0	20
	TISSEEL	14	4	18
Total		52	4	56

Chi-Square Tests

	Value	df	Asymptotic Significance (2-sided)	Exact Sig. (2-sided)
Pearson Chi-Square	9.094[a]	2	.011	**.017**
Likelihood Ratio	9.750	2	.008	.017
Fisher-Freeman-Halton Exact Test	6.575			.017
N of Valid Cases	56			

a. 3 cells (50.0%) have expected count less than 5. The minimum expected count is 1.29.

Hypothesis Test Summary

	Null Hypothesis	Test	Sig.[a,b]	Decision
1	The distribution of Weight is the same across categories of Group.	Independent-Samples Kruskal-Wallis Test	<.001	Reject the null hypothesis.
2	The distribution of Age is the same across categories of Group.	Independent-Samples Kruskal-Wallis Test	.593	Retain the null hypothesis.

a. The significance level is .050.

b. Asymptotic significance is displayed.

Group = SUTURE

Statistics[a]

		Weight	Age
N	Valid	18	18
	Missing	0	0
Median		281.00	16.00
Minimum		271	15
Maximum		297	17

a. Group = SUTURE

Group = SUTURE+TISSEEL

Statistics[a]

		Weight	Age
N	Valid	20	20
	Missing	1	1
Median		257.00	16.00
Minimum		233	15
Maximum		284	17

a. Group = SUTURE+TISSEEL

Group = TISSEEL

Statistics[a]

		Weight	Age
N	Valid	18	18
	Missing	0	0

Median	271.00	16.00
Minimum	248	15
Maximum	285	17

a. Group = TISSEEL

Crosstabs

Notes

Output Created		01-OCT-2025 11:24:55
Comments		
Input	Active Dataset	DataSet3
	Filter	<none>
	Weight	<none>
	Split File	<none>
	N of Rows in Working Data File	28
Missing Value Handling	Definition of Missing	User-defined missing values are treated as missing.
	Cases Used	Statistics for each table are based on all the cases with valid data in the specified range(s) for all variables in each table.

Syntax		CROSSTABS<br>/TABLES=Group BY Adhesions<br>/FORMAT=AVALUE TABLES<br>/STATISTICS=CHISQ<br>/CELLS=COUNT<br>/COUNT ROUND CELL<br>/METHOD=EXACT TIMER(5).
Resources	Processor Time	00:00:00,06
	Elapsed Time	00:00:00,05
	Dimensions Requested	2
	Cells Available	524245
	Time for Exact Statistics	0:00:00,01

Case Processing Summary

	Cases					
	Valid		Missing		Total	
	N	Percent	N	Percent	N	Percent
Group * Adhesions	28	100.0%	0	0.0%	28	100.0%

Group * Adhesions Crosstabulation

Count

	Adhesions	Total

		No adhesion	Thin filmy adhesion	More than one thin filmy adhesion	Thick adhesion with focal point	Thick adhesion with planar attachment	
Group	Suture	0	1	2	3	3	9
	SUTURE+TISSEEL	0	1	6	2	1	10
	TISSEEL	2	2	4	1	0	9
Total		2	4	12	6	4	28

Chi-Square Tests

	Value	df	Asymptotic Significance (2-sided)	Exact Sig. (2-sided)
Pearson Chi-Square	11.148[a]	8	.193	**.190**
Likelihood Ratio	12.225	8	.141	.303
Fisher-Freeman-Halton Exact Test	9.034			.305
N of Valid Cases	28			

a. 15 cells (100.0%) have expected count less than 5. The minimum expected count is .64.

Crosstabs

Notes

Output Created		01-OCT-2025 11:25:37
Comments		
Input	Active Dataset	DataSet3
	Filter	<none>
	Weight	<none>
	Split File	<none>
	N of Rows in Working Data File	28
Missing Value Handling	Definition of Missing	User-defined missing values are treated as missing.
	Cases Used	Statistics for each table are based on all the cases with valid data in the specified range(s) for all variables in each table.

Syntax		CROSSTABS<br>/TABLES=Group BY Adhesions_binary<br>/FORMAT=AVALUE TABLES<br>/STATISTICS=CHISQ<br>/CELLS=COUNT<br>/COUNT ROUND CELL<br>/METHOD=EXACT TIMER(5).
Resources	Processor Time	00:00:00,03
	Elapsed Time	00:00:00,03
	Dimensions Requested	2
	Cells Available	524245
	Time for Exact Statistics	0:00:00,02

Case Processing Summary

	Cases					
	Valid		Missing		Total	
	N	Percent	N	Percent	N	Percent
Group * Adhesions_binary	28	100.0%	0	0.0%	28	100.0%

Group * Adhesions_binary Crosstabulation

Count

	Adhesions_binary	Total

		No adhesions	Thin adhesions	Thick adhesions	
Group	Suture	0	3	6	9
	SUTURE+TISSEEL	0	7	3	10
	TISSEEL	2	6	1	9
Total		2	16	10	28

Chi-Square Tests

	Value	df	Asymptotic Significance (2-sided)	Exact Sig. (2-sided)
Pearson Chi-Square	9.578[a]	4	.048	**.037**
Likelihood Ratio	10.105	4	.039	.044
Fisher-Freeman-Halton Exact Test	7.721			.052
N of Valid Cases	28			

a. 6 cells (66.7%) have expected count less than 5. The minimum expected count is .64.
